# Antiparatyphoid Vaccination by the Alimentary Tract

**Published:** 1916-06

**Authors:** 


**Antiparatyphoid Vaccination by the Alimentary Tract.** J. Lignieres, Acad. of Med. of Paris, Nov. 2, 1915, described paratyphoid as an epizootic of pigs in 1900, under the name salmonella. He employed with success, virulent bacilli, killed by heat and administered in dry electuaries.

